# EHMTI-0313. Factors influencing stigma towards persons with migraine

**DOI:** 10.1186/1129-2377-15-S1-E36

**Published:** 2014-09-18

**Authors:** RE Shapiro, RB Lipton, PB Reiner

**Affiliations:** 1 Neurological Sciences, University of Vermont College of Medicine, Burlington, USA; 2 Neurology, Albert Einstein College Of Medicine, New York, USA; 3 Psychiatry, University of British Columbia, Vancouver, Canada

## Introduction

Previously, we demonstrated that stigma towards persons with migraine was comparable in magnitude to stigma towards individuals with epilepsy or panic disorder.

## Aim

We sought to measure stigma towards individuals with differing migraine phenotypes versus comparator conditions.

## Methods

Subjects were recruited on-line (MTurk), consented, and randomized to assess one of six fictional vignettes:

1) a woman with migraine four days/month with zero lost workdays/year (W0)

2) a woman with migraine four days/month with two lost workdays/year (W2)

3) a man with migraine four days/month with two lost workdays/year (M2)

4) a woman with seizures four days/month with two lost workdays/year (E2)

5) a woman caring for her invalid husband four days/month with two lost workdays/year (H2)

6) a woman with migraine twenty days/month with ten lost workdays/year (W10)

Subjects used sliders from 0 to 100 to answer five questions measuring social distance attitudes (SDA) towards the individual described in the vignette; maximum stigmatizing attitude would be a total SDA score of 500. [Study approved by UBC Office of Research Services.]

## Results

3,617 total US subjects ≥19 years old completed the survey (mean age 33.2 years; 51% female).

W2/M2/E2 scores and W0/E2 scores did not differ significantly. Relative to W2/M2, W0 was significantly lower and H2 was significantly higher. W10 was significantly higher than H2.

## Conclusions

Among MTurk subjects, stigma towards persons with migraine increased with their absenteeism, but did not vary by gender.

No conflict of interest.

